# Hemoptysis risk in hospitalized patients with bronchiectasis: development and external validation of a clinical prediction model

**DOI:** 10.3389/fmed.2026.1941626

**Published:** 2026-08-28

**Authors:** Zhijing Gong, Yi Wang, Xinxiu Liu, Jian Lu, Zhigang Cai

**Affiliations:** 1 Department of Pulmonary and Critical Care Medicine, The Second Hospital of Hebei Medical University, Shijiazhuang, Hebei, China; 2 Department of Pulmonary and Critical Care Medicine, Shijiazhuang People’s Hospital Affiliated to Hebei Medical University, Shijiazhuang, Hebei, China

**Keywords:** bronchiectasis, clinical prediction model, external validation, hemoptysis, risk prediction

## Abstract

**Background:**

Hemoptysis is a complication of bronchiectasis, but risk prediction in hospitalized patients remains insufficiently characterized. Existing bronchiectasis severity scores were developed to predict mortality, hospitalization, or exacerbations rather than hemoptysis. We aimed to develop and externally validate a model for in-hospital hemoptysis risk and interpret its predictors within prespecified clinical domains.

**Methods:**

This retrospective two-center study included 320 hospitalized adults with bronchiectasis. The derivation cohort comprised 215 patients from the Second Hospital of Hebei Medical University, and the external validation cohort comprised 105 patients from Shijiazhuang People's Hospital. Hemoptysis was the primary outcome. Candidate predictors were prespecified according to clinical relevance, clinical-domain representation, and consistency of definition and availability across centers. A core logistic regression model included age, sex, smoking history, asthma, serum albumin, hemoglobin, and log10(BNP/NT-proBNP+1); a prespecified secondary model additionally included chronic bronchitis. Discrimination, calibration, risk stratification, decision-curve analysis, and bootstrap validation were assessed.

**Results:**

Hemoptysis occurred in 55 of 215 patients (25.6%) in the derivation cohort and 36 of 105 patients (34.3%) in the validation cohort. In the core model, higher serum albumin was independently associated with hemoptysis (odds ratio per 5-g/L increase, 1.95; 95% CI, 1.29–2.94), whereas asthma showed an inverse association (odds ratio, 0.17; 95% CI, 0.06–0.51). These associations were observational and should not be interpreted as causal or protective. The core model achieved an AUC of 0.747 in the derivation cohort and 0.820 in external validation, with a Brier score of 0.177. Bootstrap validation yielded an optimism-corrected AUC of 0.707. The bronchitis-extended model achieved AUCs of 0.763 and 0.746, respectively. Across low-, intermediate-, and high-risk tertiles of the core model, observed hemoptysis rates were 2.9%, 34.3%, and 65.7%. Calibration drift was evident in the low-risk group, where the mean predicted probability was 21.4% compared with an observed rate of 2.9%.

**Conclusion:**

The core model showed good discrimination and risk stratification for in-hospital hemoptysis in bronchiectasis. The findings support a clinical-domain interpretation of bleeding susceptibility, while the albumin association should be regarded as observational. Because absolute risk was overestimated in the low-risk range, multicenter validation and local recalibration are required before probability-based clinical use.

## 1 Introduction

Non-cystic fibrosis bronchiectasis (hereafter bronchiectasis) is a chronic airway disease characterized by permanent, irreversible dilation of the bronchi, sustained by a self-perpetuating cycle of airway infection, inflammation, and progressive structural destruction (1). Its principal clinical manifestations are chronic cough, mucopurulent sputum production, and recurrent hemoptysis, frequently accompanied by airflow limitation and exertional dyspnea (1, 2). Long regarded as an orphan disease whose study has lagged behind that of chronic obstructive pulmonary disease (COPD) and asthma, bronchiectasis has shown a steadily rising recognized prevalence, with a disease burden that varies markedly across regions and is particularly high where post-infectious causes—such as prior pulmonary tuberculosis and chronic airway infection—remain common (3). Data from the Chinese Bronchiectasis Registry (BE-China) indicate that patients in China carry a substantial burden: 22.3% reported prior hemoptysis, 70.0% had at least one exacerbation and 57.2% at least one hospitalization in the preceding year, and 54.7% had severe disease according to the Bronchiectasis Severity Index (BSI), against a background of frequent cystic morphology and post-infectious or tuberculosis-related etiologies (4). Consistent with a growing emphasis on individualized risk assessment, a recent multi-registry analysis of more than 30,000 patients across European, Chinese, Indian, and Australian cohorts demonstrated that even a systemic comorbidity such as diabetes independently worsens prognosis in bronchiectasis, supporting the incorporation of comorbidities and complications into individualized risk stratification (5).

Hemoptysis is among the most clinically significant complications of bronchiectasis. Chronic airway inflammation promotes hypertrophy and tortuosity of the bronchial arteries, which arise from the high-pressure systemic circulation; rupture of these remodeled, fragile vessels is the predominant source of bleeding (6). Bronchiectasis is a common cause of hemoptysis and an important contributor to moderate-to-severe hemoptysis in particular (7). The clinical spectrum ranges from mild, self-limiting episodes to massive, life-threatening bleeding: in a US nationwide inpatient sample, hemoptysis-related hospitalizations in bronchiectasis carried an in-hospital mortality of approximately 4.5%, with about 2.1% requiring bronchial artery embolization (BAE) (8); and in severe hemoptysis the in-hospital risk of death is considerable and can be predicted from clinical and radiological variables (9). Beyond the acute hazards of airway obstruction and asphyxiation, recurrent hemoptysis can cause anemia, impair oxygenation, precipitate repeated hospitalization, and necessitate BAE, and it is associated with reduced quality of life and increased mortality (8, 9). Identifying which patients are most prone to bleed therefore has direct implications for surveillance, stable-phase management, and timely intervention.

Several validated instruments—the BSI and the FACED and E-FACED scores—have been developed and validated to grade disease severity and to predict exacerbations, hospitalization, and mortality (10–13). However, none was designed to predict hemoptysis, and their multicomponent scoring can be cumbersome to apply at the bedside. Moreover, existing studies of hemoptysis risk factors in bronchiectasis have focused almost exclusively on two specific settings: massive hemoptysis and recurrence after BAE. For massive hemoptysis, diabetes, involvement of two to three lobes, and right-upper-lobe disease have been reported as risk factors (14). For post-BAE recurrence, Pseudomonas aeruginosa—including multidrug-resistant strains—extensive bronchiectasis, increased sputum volume, abnormal bronchial arteries, cystic morphology, and post-tuberculosis destroyed lung have been repeatedly implicated (15–19); and tuberculosis and bronchiectasis are leading etiologies in major-hemoptysis cohorts characterized by high long-term recurrence (20). By contrast, the occurrence of hemoptysis in the general bronchiectasis inpatient population—that is, the clinical, laboratory, microbiological, and radiological features that distinguish patients who bleed from those who do not—remains poorly characterized. Notably, hemoptysis may not equate to more severe disease: a study comparing hemoptysis-related with infection-related hospitalizations found lower BSI and FACED scores and lower 30-day mortality in the hemoptysis group (21), suggesting that hemoptysis in bronchiectasis may represent a relatively distinct clinical phenotype rather than simply a marker of disease severity.

Previous studies of hemoptysis in bronchiectasis have mainly focused on massive hemoptysis, severe bleeding, or recurrence after bronchial artery embolization (14–20). Although these studies identified several clinical, microbiological, and structural risk factors, prediction of in-hospital hemoptysis in a broader hospitalized bronchiectasis population remains less well characterized. The present study therefore focused on this clinical setting and evaluated a parsimonious model based on routinely available variables in an independent external-center cohort without model refitting.

We therefore conducted a two-center retrospective study of hospitalized adults with bronchiectasis. Using a derivation cohort from the Second Hospital of Hebei Medical University, we aimed to identify candidate predictors associated with hemoptysis during hospitalization and to develop a clinically feasible risk model. The model was then externally validated in an independent cohort from Shijiazhuang People's Hospital Affiliated to Hebei Medical University. We further sought to interpret predictor associations within prespecified clinical domains, with particular attention to differences between infection-dominant inflammatory characteristics and hemoptysis-related clinical characteristics.

## 2 Methods

### 2.1 Study design and population

This retrospective two-center study included a derivation cohort and an independent external validation cohort of hospitalized adults with bronchiectasis admitted between January 2023 and December 2025. The derivation cohort was obtained from the Department of Pulmonary and Critical Care Medicine, the Second Hospital of Hebei Medical University, and the validation cohort from the Department of Pulmonary and Critical Care Medicine, Shijiazhuang People's Hospital Affiliated to Hebei Medical University. For patients with multiple eligible admissions, only the first hospitalization was included.

Bronchiectasis was diagnosed on the basis of compatible clinical manifestations and chest computed tomography or high-resolution computed tomography findings, including bronchial dilatation, lack of normal bronchial tapering, an increased bronchoarterial ratio, tram-track appearance, or the signet-ring sign.

The reporting of this study was structured according to the TRIPOD + AI statement for clinical prediction model studies (22); the completed checklist is provided as Supplementary Checklist S1.

### 2.2 Eligibility criteria and outcome

Patients were eligible if they were aged ≥18 years, had CT-confirmed bronchiectasis, and had sufficient information to determine eligibility and in-hospital hemoptysis status. Patients were excluded if they had cystic fibrosis-related bronchiectasis, chest trauma, thoracic deformity, congenital pulmonary malformation, or bleeding primarily attributable to causes other than bronchiectasis, including active pulmonary tuberculosis, lung abscess, pneumoconiosis, lung cancer, pulmonary embolism, systemic vasculitis, hematologic disease, severe coagulation disorders, connective tissue disease, drug- or toxin-related bleeding, or procedure-related bleeding. Hematemesis, epistaxis, and upper-airway bleeding misclassified as hemoptysis were also excluded. Stable comorbidities were not exclusion criteria. However, previous pulmonary tuberculosis and post-tuberculosis sequelae were not systematically documented, and absence of documentation could not be distinguished from true absence; these variables were therefore excluded from formal predictor analyses.

The primary outcome was hemoptysis during hospitalization, defined as expectoration of blood, blood-stained sputum, or bloody lower-respiratory secretions documented in the medical records.

### 2.3 Data collection and candidate predictors

Clinical data were extracted from electronic medical records using a standardized form. Candidate variables included demographic characteristics, smoking-related variables, airway comorbidities, hemoglobin, serum albumin, BNP or NT-proBNP, and selected inflammatory, microbiological, radiological, and cardiopulmonary findings. Laboratory measurements were obtained from the earliest available tests after admission, preferably within 24–48 h.

Candidate predictors were prespecified according to clinical relevance, representation of the proposed clinical domains, and consistency of definition and availability across centers. The domains included airway injury or reactivity, cardiopulmonary or vascular-structural burden, and hematologic or nutritional status. These domains were used only as a clinical framework for organizing and interpreting predictor associations; they were not derived through clustering, latent-class analysis, molecular profiling, or other formal phenotyping methods. Univariable *P* values were interpreted descriptively and were not used as automatic criteria for predictor inclusion or exclusion.

The core model included age, sex, smoking history, asthma, serum albumin, hemoglobin, and log10(BNP/NT-proBNP+1). A prespecified secondary bronchitis-extended model additionally included chronic bronchitis. Variables with substantial missingness, inconsistent definitions, or limited inter-center availability were not entered into the externally validated models. These included selected inflammatory, microbiological, radiological, pulmonary vascular, and morphological variables. CRP was evaluated in the derivation cohort but excluded because it reflected the acute clinical state and was not systematically collected in the validation cohort. Variable definitions, availability, model roles, and reasons for inclusion or exclusion are provided in Supplementary Table S1.

### 2.4 Statistical analysis

Continuous variables were summarized as mean ± standard deviation or median with interquartile range, and categorical variables as counts and percentages. Between-group comparisons used Welch's *t*-test, the Mann–Whitney *U*-test, or Fisher's exact test, as appropriate. Missingness was assessed separately by cohort before imputation. In the derivation cohort, age, male sex, asthma, chronic bronchitis, and hemoglobin had no missing values; serum albumin was missing in 5 patients (2.3%); and BNP/NT-proBNP was missing in 55 patients (25.6%). The exported binary smoking-history variable contained no analytical missing values, although source smoking-index documentation was unavailable for 114 patients (53.0%) and had been coded as zero. Albumin and BNP/NT-proBNP were median-imputed in the derivation cohort; male sex required no imputation. No core-model predictor required imputation in the external validation cohort. Median imputation was retained as a pragmatic deterministic approach to preserve the full derivation cohort and reduce sensitivity to extreme values in the skewed BNP/NT-proBNP distribution; however, it does not account for imputation uncertainty. Variable-specific missingness and handling are detailed in Supplementary Table S2.

Candidate predictors were prespecified according to clinical relevance, clinical-domain representation, and consistent availability across centers. Univariable logistic regression was used descriptively; *P* values were not used as automatic selection thresholds, and no forward, backward, or stepwise selection was performed. Variables with substantial missingness, inconsistent definitions, or limited inter-center availability were excluded, as detailed in Supplementary Table S1.

Two multivariable logistic regression models were fitted by maximum likelihood estimation using iteratively reweighted least squares. Age, albumin, and hemoglobin were scaled per 10 years, 5 g/L, and 10 g/L, respectively; BNP/NT-proBNP was transformed as log10(BNP/NT-proBNP+1). Multicollinearity was assessed using correlation analyses and variance inflation factors. Model performance was evaluated using AUC, calibration, Brier score, risk stratification, and decision-curve analysis. Internal validation used 1,000 bootstrap resamples. Exploratory random forest and gradient boosting models were performed to assess nonlinear feature-importance patterns but were not used for predictor selection or primary model development. Detailed machine-learning procedures are provided in the Supplementary Methods. Analyses were performed using R version 4.6.1 (R Foundation for Statistical Computing, Vienna, Austria).

### 2.4 Ethics

This study was conducted in accordance with the Declaration of Helsinki and was approved by the Research Ethics Committee of the Second Hospital of Hebei Medical University (Approval No. 2024-R333). The approval covered the retrospective use of de-identified data from both the derivation cohort and the external validation cohort at Shijiazhuang People's Hospital. The external validation center accepted the ethics approval and authorized the retrospective use of its de-identified clinical data in accordance with its institutional requirements. The requirement for written informed consent was waived under the approved protocol because of the retrospective design and the use of de-identified clinical data.

## 3 Results

### 3.1 Study population, cohort characteristics, and predictor domains

**Figure 1:**
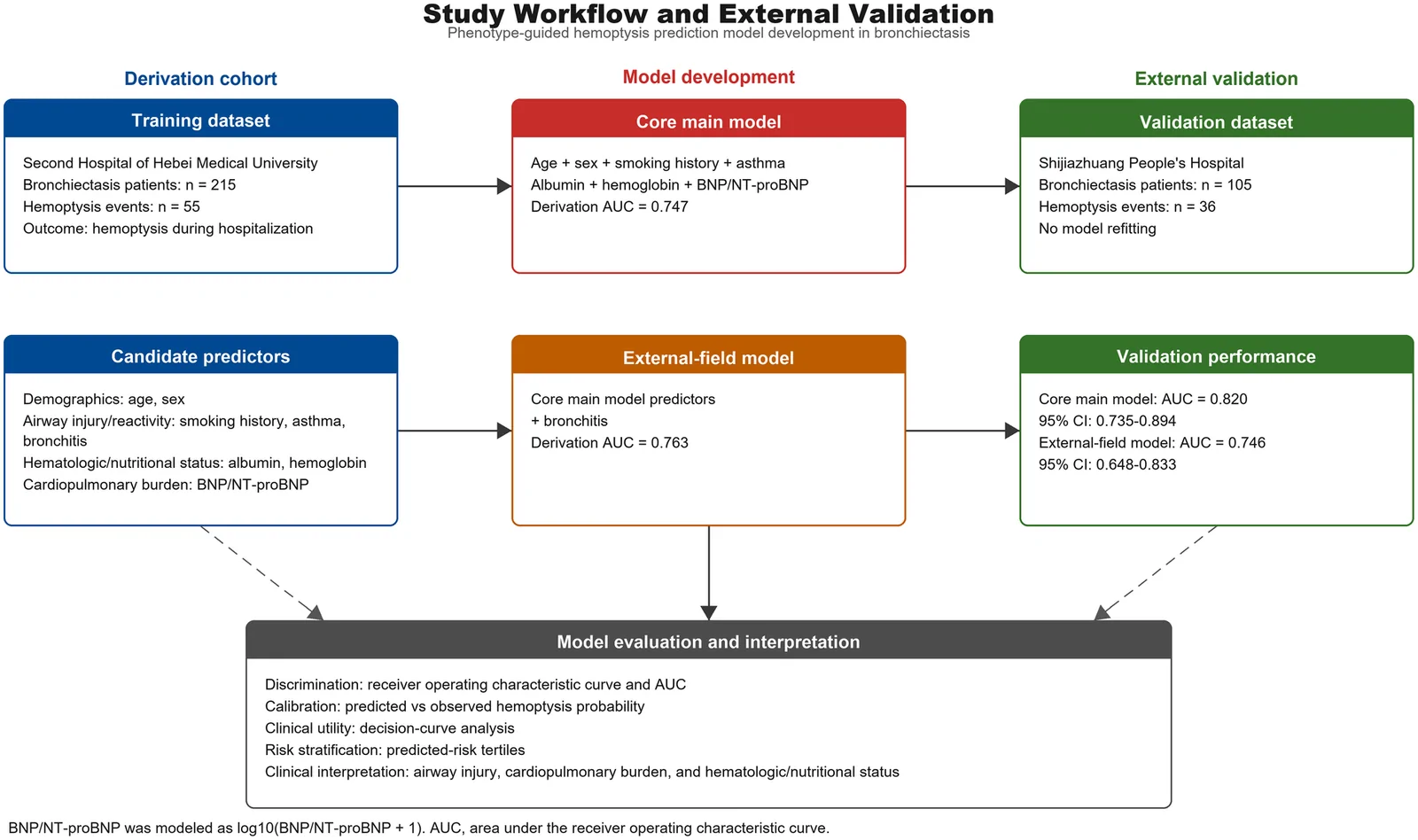
Study workflow and external validation framework. The derivation cohort included 215 patients with bronchiectasis from the Second Hospital of Hebei Medical University, including 55 hemoptysis events. Candidate predictors were organized according to demographic characteristics, airway injury or reactivity, hematologic or nutritional status, and cardiopulmonary burden. Two prespecified models were developed: the core main model and the bronchitis-extended model. External validation was performed in 105 patients from Shijiazhuang People's Hospital, including 36 hemoptysis events, without model refitting. Model performance was assessed using discrimination, calibration, decision-curve analysis, risk stratification, and clinical interpretation.

A total of 320 hospitalized adults with bronchiectasis were included. The derivation cohort comprised 215 patients from the Second Hospital of Hebei Medical University, of whom 55 (25.6%) developed hemoptysis during hospitalization. The independent external validation cohort comprised 105 patients from Shijiazhuang People's Hospital Affiliated to Hebei Medical University, of whom 36 (34.3%) developed hemoptysis. The validation cohort was used exclusively for external validation without model refitting (Figure 1).

Compared with the derivation cohort, the validation cohort had a lower prevalence of asthma (5.7% vs. 24.2%), higher mean serum albumin (40.3 vs. 36.5 g/L), and higher mean hemoglobin (130.1 vs. 123.9 g/L) (Table 1). Candidate predictors were prespecified according to clinical relevance, clinical-domain representation, and inter-center availability; full definitions and model roles are provided in Supplementary Table S1. Complete BSI and FACED scores could not be calculated because several required components were unavailable (Supplementary Table S3).

**Table 1:** Baseline characteristics of the derivation and external validation cohorts.

Variable	Derivation cohort (*n* = 215)	External validation cohort (*n* = 105)	*P* value
Age, years	60.4 ± 13.8	62.9 ± 13.4	0.115
Male sex, *n* (%)	93 (43.3)	38 (36.2)	0.276
Smoking history, *n* (%)	41 (19.1)	29 (27.6)	0.086
Asthma, *n* (%)	52 (24.2)	6 (5.7)	<0.001
Chronic bronchitis, *n* (%)	61 (28.4)	31 (29.5)	0.895
Albumin, g/L	36.5 ± 5.5	40.3 ± 3.3	<0.001
Hemoglobin, g/L	123.9 ± 19.3	130.1 ± 15.5	0.002
BNP/NT-proBNP	119.5 (62.6–186.9)	119.5 (41.0–197.0)	0.106
log10(BNP/NT-proBNP+1)	2.1 ± 0.7	2.0 ± 0.5	0.071
Hemoptysis during hospitalization, *n* (%)	55 (25.6)	36 (34.3)	0.114

Values are reported from the analysis dataset as mean ± SD, median (interquartile range), or *n* (%). Between-cohort *P* values are descriptive and were calculated using Welch's *t*-test for normally distributed continuous variables, the Mann–Whitney U test for BNP/NT-proBNP, and Fisher's exact test for categorical variables. In the derivation cohort, missing albumin values (5/215, 2.3%) and BNP/NT-proBNP values (55/215, 25.6%) were replaced using median imputation. Derivation-cohort smoking history was derived from the exported smoking index; limitations in source documentation and variable-specific missingness are detailed in Supplementary Table S2. BNP, B-type natriuretic peptide; NT-proBNP, N-terminal pro-B-type natriuretic peptide.

### 3.2 Univariable findings and multivariable model development

Univariable logistic regression results are shown in Table 2. Younger age, absence of asthma, higher albumin, lower CRP, and lower WBC were associated with hemoptysis, while chronic bronchitis and hemoglobin showed borderline associations. Univariable *P* values were interpreted descriptively and were not used for predictor selection.

**Table 2:** Univariable analysis of factors associated with hemoptysis in the derivation cohort.

Variable	Unit/comparison	Hemoptysis (*n* = 55)	No hemoptysis (*n* = 160)	OR (95% CI)	*P* value
Age	Per 10-year increase	56.2 ± 15.0	61.8 ± 13.1	0.75 (0.61–0.94)	0.011
Male sex	Male vs. female	29 (52.7)	64 (40.0)	1.67 (0.90–3.10)	0.102
Smoking history	Yes vs. no	13 (23.6)	28 (17.5)	1.46 (0.69–3.07)	0.319
Asthma	Yes vs. no	4 (7.3)	48 (30.0)	0.18 (0.06–0.53)	0.002
Chronic bronchitis	Yes vs. no	10 (18.2)	51 (31.9)	0.47 (0.22–1.02)	0.055
Albumin	Per 5-g/L increase	38.8 ± 5.4	35.7 ± 5.3	1.80 (1.28–2.53)	<0.001
Hemoglobin	Per 10-g/L increase	128.2 ± 18.3	122.4 ± 19.4	1.17 (1.00–1.38)	0.056
log10(BNP/NT-proBNP+1)	Per 1-log10 increase	2.1 ± 0.6	2.1 ± 0.7	0.87 (0.54–1.40)	0.561
CRP	Per 10-unit increase	6.9 (1.8–12.2)	13.1 (3.5–48.2)	0.91 (0.83–0.99)	0.031
WBC	Per 1-unit increase	6.3 ± 2.3	7.4 ± 3.6	0.88 (0.79–0.99)	0.035
NLR	Per 1-unit increase	3.2 (2.3–4.0)	3.8 (2.6–5.9)	0.96 (0.87–1.05)	0.362
Multilobar involvement	Yes vs. no	22 (40.0)	72 (45.0)	0.81 (0.44–1.52)	0.519

Odds ratios (ORs) and 95% confidence intervals (CIs) were estimated using univariable logistic regression, with continuous predictors scaled as specified. Wald *P* values are reported. Values are presented as mean ± SD, median (interquartile range), or *n* (%). CRP and NLR are presented as median (interquartile range) because of their skewed distributions. The regression *P* values were derived from univariable logistic regression rather than from the descriptive between-group comparisons. Associations should not be interpreted as causal or protective effects. CRP, C-reactive protein; WBC, white blood cell count; NLR, neutrophil-to-lymphocyte ratio.

The core model included age, sex, smoking history, asthma, albumin, hemoglobin, and log10(BNP/NT-proBNP+1). The prespecified secondary bronchitis-extended model additionally included chronic bronchitis. In the core model, albumin was associated with higher odds of hemoptysis per 5-g/L increase (OR, 1.95; 95% CI, 1.29–2.94; *P* = 0.002), whereas asthma showed an inverse association (OR, 0.17; 95% CI, 0.06–0.51; *P* = 0.002). Other predictors were not statistically significant (Table 3).

**Table 3:** Multivariable logistic regression models in the derivation cohort.

Model	Predictor	Coding/scaling	*β*	SE	OR (95% CI)	*P* value
Core model	Age	Per 10-year increase	−0.207	0.126	0.81 (0.64–1.04)	0.100
Core model	Male sex	Male vs. female	0.656	0.445	1.93 (0.80–4.61)	0.141
Core model	Smoking history	Yes vs. no	0.098	0.496	1.10 (0.42–2.92)	0.844
Core model	Asthma	Yes vs. no	−1.777	0.565	0.17 (0.06–0.51)	0.002
Core model	Albumin	Per 5-g/L increase	0.666	0.211	1.95 (1.29–2.94)	0.002
Core model	Hemoglobin	Per 10-g/L increase	−0.081	0.111	0.92 (0.74–1.15)	0.463
Core model	log10(BNP/NT-proBNP+1)	Per 1-log10 increase	0.132	0.287	1.14 (0.65–2.00)	0.645
Bronchitis-extended model	Age	Per 10-year increase	−0.142	0.131	0.87 (0.67–1.12)	0.280
Bronchitis-extended model	Male sex	Male vs. female	0.530	0.455	1.70 (0.70–4.14)	0.244
Bronchitis-extended model	Smoking history	Yes vs. no	0.127	0.506	1.13 (0.42–3.06)	0.802
Bronchitis-extended model	Asthma	Yes vs. no	−1.852	0.569	0.16 (0.05–0.48)	0.001
Bronchitis-extended model	Chronic bronchitis	Yes vs. no	−0.885	0.433	0.41 (0.18–0.96)	0.041
Bronchitis-extended model	Albumin	Per 5-g/L increase	0.690	0.217	1.99 (1.30–3.05)	0.002
Bronchitis-extended model	Hemoglobin	Per 10-g/L increase	−0.046	0.114	0.96 (0.76–1.19)	0.685
Bronchitis-extended model	log10(BNP/NT-proBNP+1)	Per 1-log10 increase	0.056	0.292	1.06 (0.60–1.87)	0.848

The prespecified core model included age, sex, smoking history, asthma, albumin, hemoglobin, and log10(BNP/NT-proBNP+1); the bronchitis-extended model additionally included chronic bronchitis. β denotes the logistic regression coefficient and SE its standard error. Positive albumin and inverse asthma/chronic bronchitis coefficients represent adjusted associations and do not imply that increasing albumin causes bleeding or that airway-disease diagnoses are causally protective. Intercepts, complete formulas, and reproducible coding are provided in Supplementary Table S6.

Among patients with observed albumin measurements before imputation, median albumin was 39.7 g/L (IQR, 36.9–41.1) in the hemoptysis group and 36.3 g/L (IQR, 32.0–40.1) in the non-hemoptysis group. Hypoalbuminemia (<35 g/L) was present in 18.9% and 42.0%, respectively (*P* = 0.003; Supplementary Table S4). In the bronchitis-extended model, chronic bronchitis showed an inverse association (OR, 0.41; 95% CI, 0.18–0.96; *P* = 0.041), while albumin (OR, 1.99; 95% CI, 1.30–3.05; *P* = 0.002) and asthma (OR, 0.16; 95% CI, 0.05–0.48; *P* = 0.001) remained associated. These findings were observational, not causal or protective.

Pearson and Spearman correlation analyses did not indicate substantial collinearity (Supplementary Figures S1, S2). The maximum variance inflation factor was 1.71 in the core model and 1.72 in the bronchitis-extended model, with all tolerance values >0.50 (Supplementary Table S5). Complete predictor coding, coefficients, intercepts, and equations are provided in Supplementary Table S6.

### 3.3 Model discrimination and internal validation

**Figure 2:**
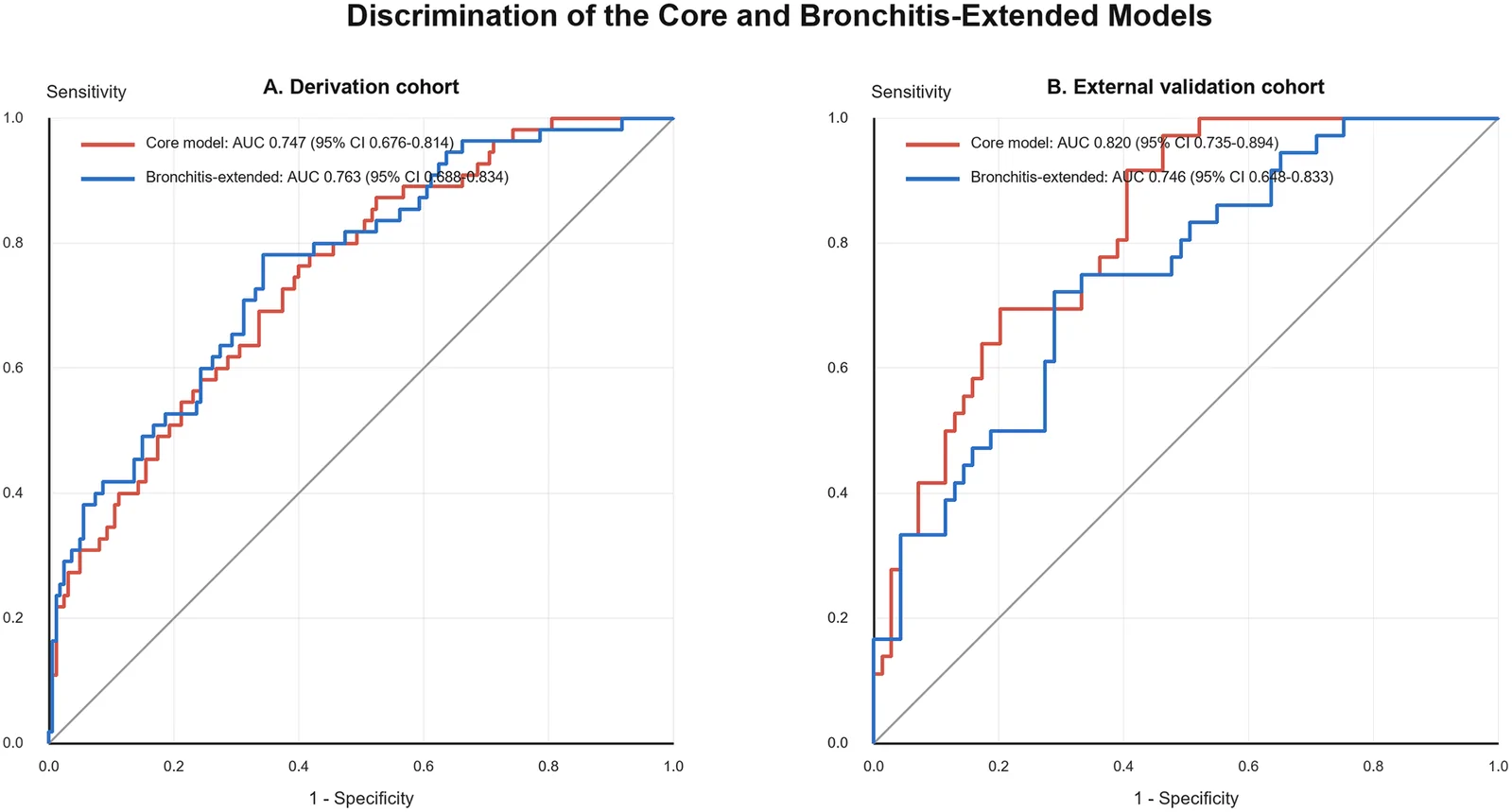
Discrimination of the core and bronchitis-extended models. Receiver operating characteristic curves show the discrimination of the core model and bronchitis-extended model in **(A)** the derivation cohort and **(B)** the external validation cohort. The core model achieved an AUC of 0.747 in the derivation cohort and 0.820 in the external validation cohort. The bronchitis-extended model achieved an AUC of 0.763 in the derivation cohort and 0.746 in the external validation cohort. AUC, area under the receiver operating characteristic curve.

Model discrimination and external calibration metrics are summarized in Table 4A, and the corresponding receiver operating characteristic curves are shown in Figure 2. In the derivation cohort, the core and bronchitis-extended models achieved AUCs of 0.747 (95% CI, 0.676–0.814) and 0.763 (95% CI, 0.688–0.834), respectively. In external validation, the core model achieved an AUC of 0.820 (95% CI, 0.735–0.894) and a Brier score of 0.177, whereas the bronchitis-extended model achieved an AUC of 0.746 (95% CI, 0.648–0.833) and a Brier score of 0.187.

**Table 4:** Model performance and external risk stratification. A Discrimination and calibration.

Model	Derivation AUC (95% CI)	External AUC (95% CI)	External Brier score	Calibration intercept (95% CI)	Calibration slope (95% CI)
Core model	0.747 (0.676–0.814)	0.820 (0.735–0.894)	0.177	0.725 (0.012–1.438)	2.773 (1.513–4.033)
Bronchitis-extended model	0.763 (0.688–0.834)	0.746 (0.648–0.833)	0.187	0.134 (−0.430–0.698)	1.688 (0.787–2.589)

**Table:** B External risk stratification by predicted-risk tertiles.

Model	Risk stratum	n	Events, n	Observed hemoptysis rate	Mean predicted probability
Core model	Low	35	1	2.9%	21.4%
Core model	Intermediate	35	12	34.3%	35.7%
Core model	High	35	23	65.7%	51.0%
Bronchitis-extended model	Low	35	5	14.3%	20.7%
Bronchitis-extended model	Intermediate	35	13	37.1%	37.6%
Bronchitis-extended model	High	35	18	51.4%	53.6%

External validation was performed in the Shijiazhuang People's Hospital cohort without model refitting. Panel A summarizes discrimination and external calibration, whereas Panel B summarizes external risk stratification according to model-predicted tertiles. Calibration intercept and slope were estimated by logistic recalibration of the external outcome on the original linear predictor; ideal values are 0 and 1, respectively. Risk strata were defined separately for each model. AUC, area under the receiver operating characteristic curve; CI, confidence interval.

With seven predictor parameters and 55 events, the core model had 7.9 events per predictor parameter. Bootstrap validation using 1,000 resamples estimated an optimism of 0.040. The optimism-corrected AUC was 0.707, and the corrected calibration slope was 0.786 **(**Supplementary Table S7). Primary interpretation of subsequent calibration and risk-stratification analyses focused on the core model because it showed better external discrimination.

### 3.4 External calibration, risk stratification, and clinical utility

**Figure 3:**
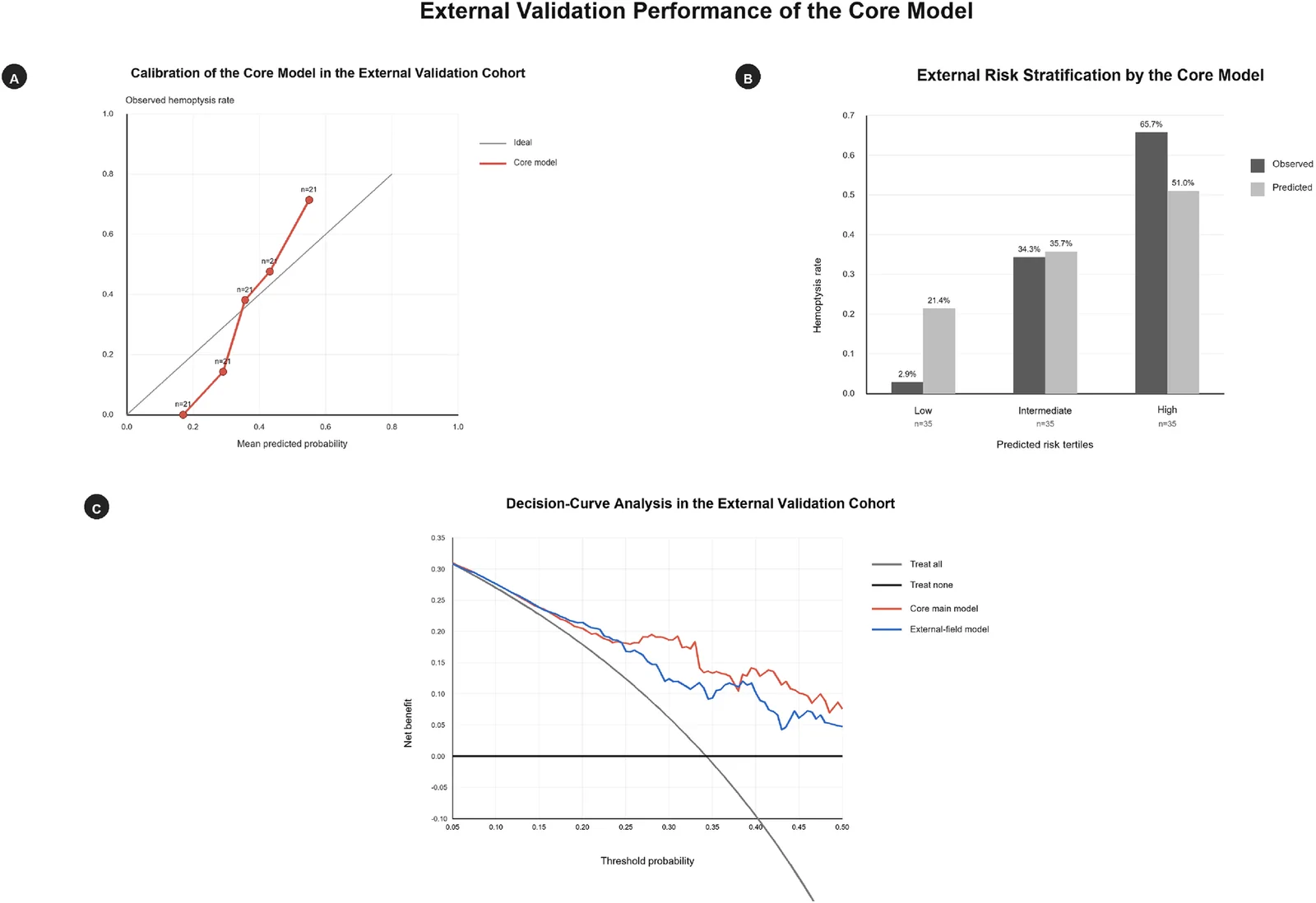
External validation performance and clinical utility of the prediction models. **(A)** Calibration plot of the core model in the external validation cohort. The model showed calibration drift, particularly in the low-risk range, where predicted probabilities exceeded observed hemoptysis rates. **(B)** Risk stratification of the core model according to tertiles of predicted risk. Observed hemoptysis rates were 2.9%, 34.3%, and 65.7% in the low-, intermediate-, and high-risk groups, respectively. **(C)** Decision-curve analysis of the core and bronchitis-extended models in the external validation cohort.

The core model showed calibration drift, particularly in the low-risk range (Figure 3A). The calibration intercept was 0.725 (95% CI, 0.012–1.438), and the calibration slope was 2.773 (95% CI, 1.513–4.033). Across low-, intermediate-, and high-risk tertiles, observed hemoptysis rates were 2.9%, 34.3%, and 65.7%, compared with mean predicted probabilities of 21.4%, 35.7%, and 51.0%, respectively (Table 4B and Figure 3B). Thus, risk ranking was preserved despite overestimation in the low-risk group.

For the bronchitis-extended model, the calibration intercept was 0.134 (95% CI, −0.430–0.698), and the calibration slope was 1.688 (95% CI, 0.787–2.589) (Table 4A and Supplementary Figure S3). Across the low-, intermediate-, and high-risk tertiles, observed hemoptysis rates were 14.3%, 37.1%, and 51.4%, compared with mean predicted probabilities of 20.7%, 37.6%, and 53.6%, respectively (Table 4B and Supplementary Figure S4). Additional risk-stratification and calibration results are provided in Supplementary Tables S8, S9.

In descriptive model-updating analyses, intercept-only updating of the core model yielded a Brier score of 0.178, whereas recalibration of both the intercept and slope reduced the Brier score to 0.163. These same-cohort analyses were descriptive and did not constitute an additional independent validation (Supplementary Table S9). Decision-curve analysis showed greater net benefit than treat-all or treat-none strategies across selected threshold probabilities (Figure 3C).

### 3.5 Model interpretation and clinical-domain framework

**Figure 4:**
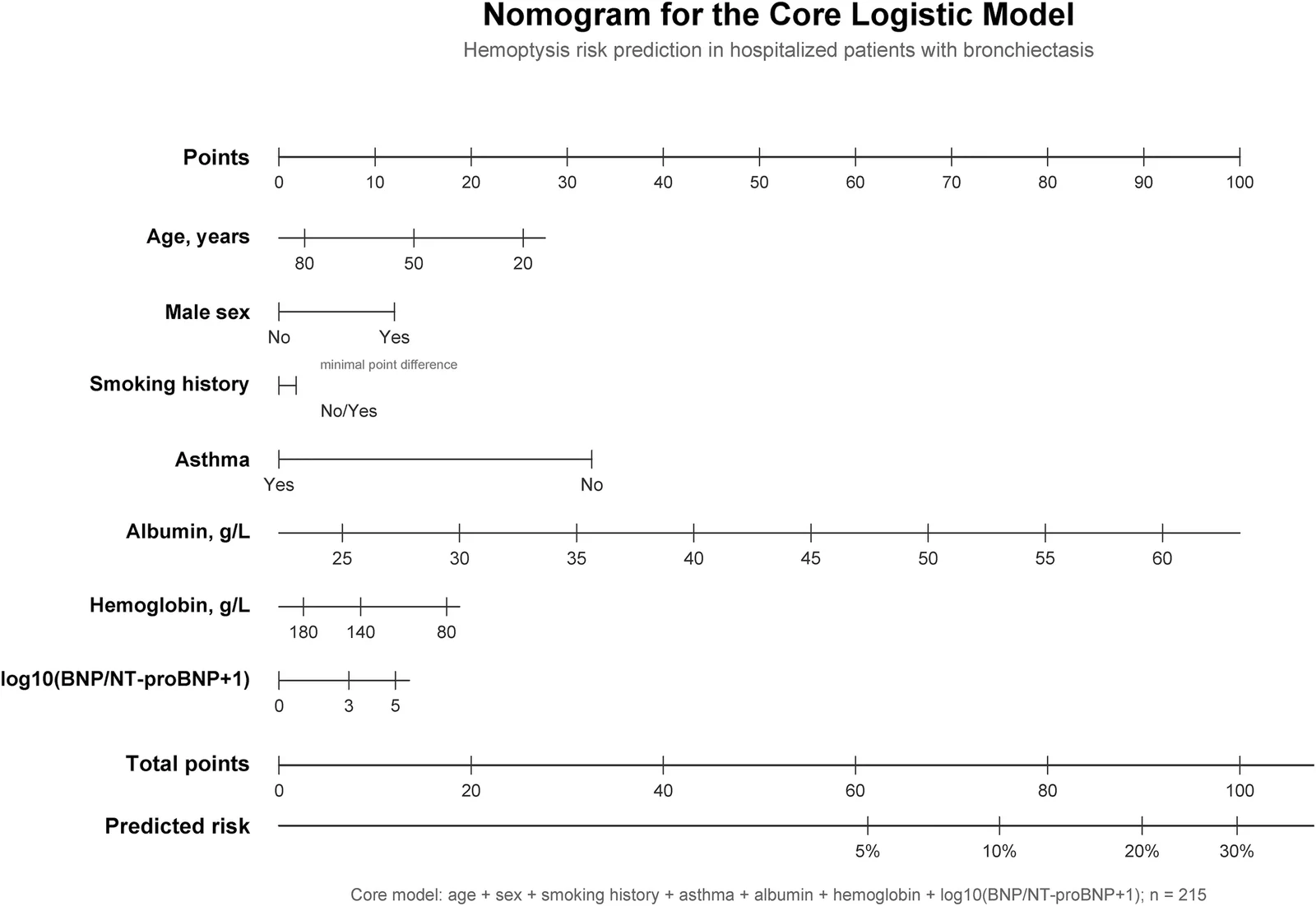
Nomogram for the core logistic regression model. The nomogram was constructed from the core multivariable logistic regression model developed in the derivation cohort. Points are assigned according to age, sex, smoking history, asthma, serum albumin, hemoglobin, and log10(BNP/NT-proBNP+1). The points for all predictors are summed to obtain the total score, which is then mapped to the estimated probability of in-hospital hemoptysis. Because external validation showed calibration drift, particularly in the low-risk range, the nomogram should be used primarily for risk stratification rather than direct estimation of individual absolute risk without local recalibration. BNP, B-type natriuretic peptide; NT-proBNP, N-terminal pro-B-type natriuretic peptide.

A nomogram based on the core model is presented in Figure 4. Given the calibration drift, it is intended for reproducible calculation and risk stratification rather than direct absolute-risk estimation without local recalibration.

Exploratory random forest and gradient boosting analyses were restricted to the derivation cohort. Mean cross-validated AUCs were 0.731 (SD, 0.053) and 0.671 (SD, 0.081), respectively. Asthma and albumin showed the largest mean permutation importance in both models. Smoking history was included; its mean decrease in validation AUC was 0.0067 for random forest and approximately 0 for gradient boosting. Several predictors showed near-zero or negative importance. These analyses were not externally validated and were not used for primary predictor selection or causal interpretation (Supplementary Tables S10, S11, Figure S5).

**Figure 5:**
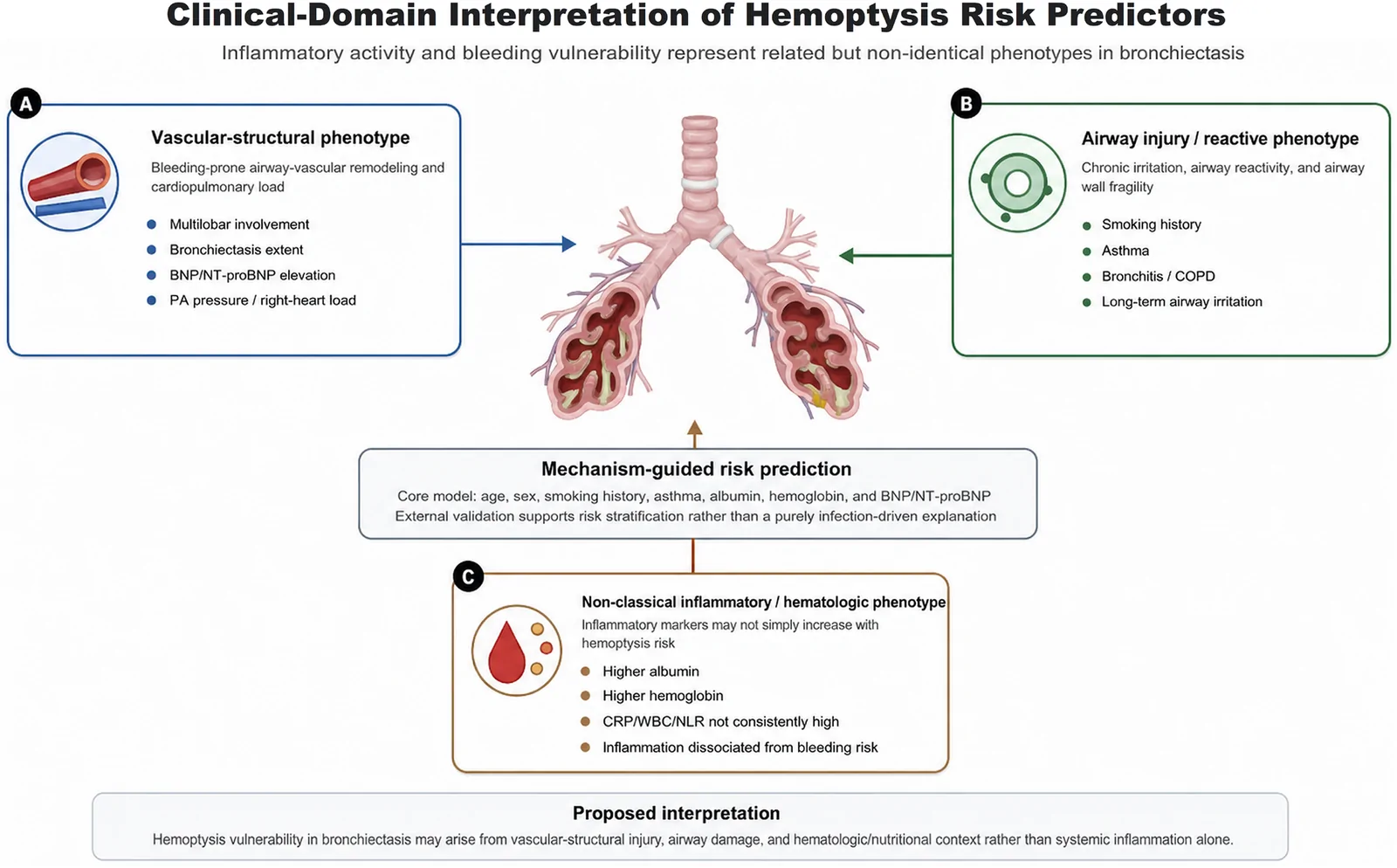
Clinical-domain framework for interpreting hemoptysis risk in bronchiectasis. The proposed framework summarizes three clinically relevant domains associated with hemoptysis vulnerability: **(A)** vascular-structural or cardiopulmonary burden, **(B)** airway injury or reactivity, and **(C)** hematologic, nutritional, or non-classical inflammatory status. These domains suggest that hemoptysis risk may reflect interacting vascular, airway, cardiopulmonary, and systemic features rather than systemic inflammation alone. The framework is descriptive and interpretive, does not represent formal phenotyping, and does not imply causal effects of individual predictors.

The clinical-domain framework is shown in Figure 5. The predictors were organized into airway injury or reactivity, cardiopulmonary or vascular-structural burden, and hematologic or nutritional status. This framework was used to support clinical interpretation of predictor associations and was not intended to define formal bronchiectasis phenotypes.

## 4 Discussion

In this two-center study of hospitalized patients with bronchiectasis, we developed and externally validated a clinically accessible model for in-hospital hemoptysis risk. Hemoptysis occurred in 25.6% of the derivation cohort and 34.3% of the external validation cohort, consistent with previous bronchiectasis cohorts and hemoptysis-related hospitalization studies (4, 8, 21). The core model, including age, sex, smoking history, asthma, serum albumin, hemoglobin, and log10(BNP/NT-proBNP+1), achieved an AUC of 0.747 in the derivation cohort and 0.820 in the external validation cohort, and stratified patients into distinct risk groups. Unlike BSI, FACED, and E-FACED scores, which were primarily developed for disease severity, exacerbation, hospitalization, or mortality prediction, this model specifically addressed in-hospital hemoptysis risk and underwent independent external validation (10–13). Model performance should therefore be interpreted considering discrimination, calibration, and clinical applicability rather than discrimination alone (22, 23). The main contribution of the present study lies in its focus on in-hospital hemoptysis in a general hospitalized bronchiectasis population, together with independent external validation and explicit assessment of calibration, risk stratification, and clinical utility.

Our findings suggest that bronchiectasis-associated hemoptysis is not simply a manifestation of infection-dominant airway inflammation. Although bronchiectasis is an important cause of hemoptysis ranging from mild bleeding to life-threatening events requiring BAE or associated with recurrence risk (6–9), previous studies indicate that hemoptysis-related hospitalization may differ from infection-related exacerbation. Seo et al. reported that bronchiectasis patients hospitalized with hemoptysis did not have higher BSI or FACED scores or mortality than those hospitalized for infection-related exacerbations, whereas previous tuberculosis, mycetoma, and bronchial artery hypertrophy were associated with hemoptysis (21). Consistently, CRP, WBC, and NLR did not show a consistent positive association with hemoptysis in our cohort, suggesting that bleeding susceptibility and inflammatory activity may represent overlapping but distinct clinical dimensions.

The inverse associations of asthma and chronic bronchitis should be interpreted as contrasting clinical patterns rather than protective effects. One possible explanation is the presence of contrasting admission patterns within hospitalized bronchiectasis populations. Patients dominated by chronic cough, sputum production, mucus hypersecretion, and infection-related airway inflammation may be admitted mainly because of an infection-dominant airway pattern, whereas patients admitted with hemoptysis may be relatively enriched for vascular-structural bleeding susceptibility (21, 25). These patterns are not mutually exclusive and do not indicate that asthma or chronic bronchitis prevents bleeding. Instead, the observed associations may partly reflect differences in reasons for admission, inpatient case mix, and retrospective selection. Furthermore, chronic bronchitis is a diagnosis-label variable that may be influenced by center-specific diagnostic criteria and documentation practices, which may explain the reduced external performance of the bronchitis-extended model. Asthma may similarly represent an airway-reactivity pattern rather than a direct determinant of bleeding risk (1, 2, 24, 26).

The association between serum albumin and hemoptysis requires cautious interpretation. Although each 5-g/L increase in albumin was independently associated with higher odds of hemoptysis, this finding should not be interpreted as evidence that albumin directly promotes bleeding or that increasing albumin levels would increase hemoptysis risk. In the observed-value analysis, median albumin was higher in the hemoptysis group, whereas hypoalbuminemia was more common in the non-hemoptysis group. This pattern suggests that the association may partly reflect relative hypoalbuminemia among patients hospitalized without hemoptysis rather than pathological albumin elevation among those with hemoptysis. Albumin may decrease during systemic inflammation, increased capillary permeability, catabolism, and nutritional deterioration (27), and lower albumin has also been associated with respiratory hospitalization in patients with bronchiectasis (28). Patients hospitalized primarily for infection-related exacerbations may therefore have lower albumin concentrations, whereas hemoptysis itself may prompt admission among patients with relatively preserved systemic status. Albumin should consequently be interpreted as an observational marker of systemic clinical state rather than a causal determinant.

The proposed predictor domains provide a clinical framework for understanding hemoptysis vulnerability. The vascular-structural domain is supported by previous evidence that bronchial artery hypertrophy, abnormal systemic collaterals, structural destruction, cystic bronchiectasis, post-tuberculosis changes, and Pseudomonas infection are associated with severe or recurrent hemoptysis (6, 14–21, 29–31). Although detailed vascular imaging was unavailable in our cohort, these factors support the biological relevance of vascular and structural susceptibility. The airway injury or reactivity domain reflects the heterogeneous nature of bronchiectasis, in which airway infection, inflammation, mucus dysfunction, and structural damage interact over time (1–3, 24, 32, 33). The hematologic, nutritional, and cardiopulmonary domain further indicates that hemoptysis risk cannot be characterized solely by conventional inflammatory markers. Registry studies have demonstrated that bronchiectasis outcomes are influenced by etiology, comorbidities, and systemic status (4, 5).

The bronchitis-extended model showed slightly better discrimination in the derivation cohort but inferior performance during external validation. Chronic bronchitis was derived from clinical documentation and may vary according to diagnostic criteria, coding practices, and overlap with COPD or bronchiectasis-related sputum production. Therefore, its predictive contribution may not be sufficiently transportable across institutions. The more parsimonious core model, based mainly on routinely available demographic and laboratory variables, may provide better generalizability.

External validation revealed meaningful differences in case mix between the two centers. Compared with the derivation cohort, the validation cohort had a substantially lower prevalence of asthma (5.7% vs. 24.2%) and higher mean albumin (40.3 vs. 36.5 g/L) and hemoglobin levels (130.1 vs. 123.9 g/L). Because asthma carried an inverse coefficient and albumin a positive coefficient in the core model, the lower prevalence of asthma and higher albumin levels in the validation cohort tended to shift predicted risks upward. This difference in predictor distribution may have contributed to the observed overestimation in the low-risk range. Higher hemoglobin, which had a small inverse coefficient in the model, may have partly offset this effect, although its estimated association was weak. Importantly, the validation cohort also had a higher overall hemoptysis rate (34.3% vs. 25.6%), indicating that calibration drift cannot be explained by a single predictor or by baseline event frequency alone. Differences in case mix, predictor distributions, admission patterns, and center-specific documentation may therefore have altered both baseline risk and the transportability of predictor effects. The model should therefore currently be considered a risk-stratification tool rather than a method for estimating individual absolute probabilities. Larger prospective multicenter validation and local recalibration are required before probability-based clinical implementation. Decision-curve analysis suggested potential clinical utility, but these findings should support closer assessment and monitoring rather than directly guide invasive procedures such as BAE (22, 23, 34, 35).

Several limitations should be acknowledged. First, the derivation cohort included only 55 hemoptysis events for seven predictor parameters, corresponding to approximately 7.9 events per parameter. The limited number of events increases uncertainty in the estimated regression coefficients and may contribute to model optimism and coefficient instability. Bootstrap validation indicated some optimism, with the apparent AUC decreasing from 0.747 to an optimism-corrected value of 0.707 and a corrected calibration slope of 0.786. Although independent external validation provides additional information on transportability, it does not eliminate uncertainty in the original coefficient estimates. The model should therefore be regarded as preliminary, and larger prospective multicenter cohorts are needed to assess coefficient stability, generalizability, and the need for model updating. Second, external calibration was imperfect, particularly in the low-risk group, in which the original model overestimated hemoptysis probability. The model is therefore currently more suitable for relative risk stratification than for estimating individual absolute probabilities, and local intercept updating or recalibration may be required before probability-based clinical implementation. Third, several clinically relevant variables, including microbiological, radiological, pulmonary vascular, and detailed structural features, were unavailable or inconsistently recorded across centers. Previous tuberculosis and post-tuberculosis sequelae could not be reliably evaluated, and incomplete data prevented direct comparison with the BSI and FACED scores (10–13). Source smoking-index documentation was incomplete in the derivation cohort, and undocumented values had been coded as zero in the exported dataset; potential misclassification of smoking history may therefore have affected its estimated association. The use of single median imputation, particularly for BNP/NT-proBNP, does not account for imputation uncertainty and represents an additional methodological limitation. Fourth, the observed associations of albumin, asthma, and chronic bronchitis may partly reflect inpatient case mix, residual confounding, and center-specific documentation practices rather than causal relationships. Finally, hemoptysis was evaluated as a binary in-hospital outcome, without systematic assessment of bleeding volume, severity, recurrence, or BAE-related outcomes (14–21).

In conclusion, this study developed and externally validated a clinically accessible model for in-hospital hemoptysis risk in bronchiectasis. The core model showed good external discrimination and identified clinically distinct risk groups. The findings support a clinical-domain interpretation of hemoptysis vulnerability, although individual predictor associations should be interpreted cautiously. Further prospective multicenter validation and local recalibration are required before probability-based clinical implementation.

## Data Availability

The raw data supporting the conclusions of this article will be made available by the authors, without undue reservation.
