# Supplementary material for: Hemoptysis risk in hospitalized patients with bronchiectasis: development and external validation of a clinical prediction model
Source: Front Med (Lausanne). 2026 Aug 28;13:1941626. doi: 10.3389/fmed.2026.1941626 (PMC13561999; doi:10.3389/fmed.2026.1941626)
Supplement: Supplementary file 1 [file Supplementaryfile1.docx]

**Supplementary Methods**

**S1. Reporting guideline**

This study was reported in accordance with the TRIPOD+AI statement for studies developing and evaluating clinical prediction models. The completed TRIPOD+AI checklist is provided as Supplementary Checklist S1.

**S2. Data preprocessing and missing-data handling**

Candidate predictors were harmonized across cohorts before model development. Binary predictors were coded as 1 when present and 0 when absent. Age, albumin, and hemoglobin were analyzed per 10 years, 5 g/L, and 10 g/L, respectively. BNP/NT-proBNP was transformed as log10(BNP/NT-proBNP + 1) because of its right-skewed distribution.

Missingness was assessed before imputation in each cohort. In the derivation cohort, albumin was missing in 5 of 215 patients (2.3%), and BNP/NT-proBNP in 55 (25.6%). Age, male sex, asthma, chronic bronchitis, and hemoglobin had no missing values. The exported binary smoking-history variable contained no analytical missing values because unavailable source smoking-index records for 114 patients (53.0%) had previously been coded as zero. Thus, undocumented smoking status could not be distinguished from an explicitly recorded absence of smoking history.

Male sex was imputed using the mode, whereas albumin and BNP/NT-proBNP were imputed using the derivation-cohort median. Median imputation was selected as a pragmatic deterministic approach to retain the complete cohort and reduce the influence of extreme BNP/NT-proBNP values. This approach does not account for imputation uncertainty and is acknowledged as a limitation. No final-model predictor required imputation in the external validation cohort. Detailed predictor-level missingness and handling are reported in Supplementary Table S2.

**S3. Development of the prespecified logistic regression models**

The core model included age, male sex, smoking history, asthma, albumin, hemoglobin, and log10(BNP/NT-proBNP + 1). The bronchitis-extended model additionally included chronic bronchitis. Predictor inclusion was prespecified on the basis of clinical relevance and inter-center availability; stepwise variable selection was not performed.

Models were fitted using maximum-likelihood logistic regression with a logit link and an iteratively reweighted least-squares algorithm, as implemented by the glm function in R. The complete intercepts, regression coefficients, predictor coding, odds ratios, confidence intervals, and equations are provided in Supplementary Table S6.

**S4. Bootstrap internal validation**

Internal validation of the core model used 1,000 bootstrap resamples. In each resample, the model was refitted in the bootstrap sample and evaluated in both the bootstrap sample and the original derivation cohort. Mean optimism was calculated as the difference between these two estimates and subtracted from the apparent performance. The calibration slope was corrected using the same optimism-estimation framework. The random seed was 20260801.

The core model was developed from 55 hemoptysis events and seven predictor parameters, corresponding to 7.9 events per parameter. Bootstrap validation estimates model optimism but does not eliminate uncertainty arising from the limited event count; coefficient instability, overfitting, and reduced transportability therefore remain possible.

**S5. External validation, calibration, and clinical utility**

The original model intercepts and coefficients were applied unchanged to the external validation cohort; no model refitting or predictor re-estimation was performed. Discrimination was quantified using the area under the receiver operating characteristic curve with 95% percentile-bootstrap confidence intervals. Overall prediction error was summarized using the Brier score.

Calibration was evaluated graphically across quintiles of predicted probability and by estimating the calibration intercept and slope from logistic recalibration. Risk stratification was examined across model-specific tertiles of predicted probability. Decision-curve analysis compared model-guided strategies with treat-all and treat-none strategies across clinically relevant threshold probabilities.

**S6. Exploratory machine-learning analyses**

Random forest and gradient boosting models were conducted as exploratory secondary analyses to examine potential nonlinear associations and feature-importance patterns in the derivation cohort. These analyses were restricted to the derivation cohort and were not used for predictor selection, development of the prespecified logistic regression models, external validation, or interpretation of individual absolute risk.

Both models included age, sex, smoking history, asthma, chronic bronchitis, chronic obstructive pulmonary disease, albumin, hemoglobin, log10(BNP/NT-proBNP + 1), pulmonary artery pressure, multilobar involvement, bronchiectasis extent, C-reactive protein, white blood cell count, and neutrophil-to-lymphocyte ratio. Smoking history was explicitly included as a binary predictor and was defined from the exported smoking index as an index greater than zero. In the derivation dataset, unavailable source smoking-index values had previously been coded as zero; therefore, the exploratory machine-learning results involving smoking history should be interpreted in light of this source-data limitation.

Continuous predictors were retained on their original scales except BNP/NT-proBNP, which was transformed as log10(BNP/NT-proBNP + 1); binary predictors were coded as 0 or 1. No standardization was applied because both algorithms were tree based. Within every resampling iteration, missing continuous values were replaced by the median and missing binary values by the mode estimated from the corresponding training fold; these values were then applied to the validation fold to prevent information leakage. Class imbalance was addressed using inverse-frequency class weights for random forest and inverse-frequency observation weights for gradient boosting.

Analyses were performed in R version 4.6.1 using the randomForest package (version 4.7-1.2) and the gbm package (version 2.3.1). Hyperparameters were selected by grid search with ROC AUC as the optimization metric. Internal performance was estimated using nested stratified five-fold cross-validation repeated five times. Four-fold cross-validation within each outer training set was used for hyperparameter selection, and performance was evaluated in the held-out outer fold. Prespecified random seeds were 20260802 for random forest and 20270802 for gradient boosting. Search spaces and selected hyperparameters are reported in Supplementary **Table S10**.

Permutation importance was calculated in each held-out outer validation fold. Each predictor was permuted 20 times, and importance was defined as the mean decrease in validation AUC relative to the unpermuted predictions. Values were averaged across the 25 outer validation folds. All input variables were retained in the display, including variables with zero, near-zero, or negative importance. The complete values are provided in Supplementary **Table S11** and visualized in **Supplementary Figure S5**.

**Supplementary Tables**

**Supplementary Table S1. Candidate predictors, clinical-domain grouping, coding, availability, and model role.**

| **Variable** | **Clinical domain** | **Definition/coding** | **Scaling** | **Availability** | **Model role** | **Reason for inclusion or exclusion** |
| --- | --- | --- | --- | --- | --- | --- |
| Age | Demographic | Age at hospitalization, years | Per 10 years | Both cohorts | Core; extended | Clinically relevant and consistently available |
| Male sex | 0 (0.0%) | Male=1; female=0 | No imputation required in either cohort | Both cohorts | Core; extended | Clinically relevant and consistently available |
| Smoking history | Airway injury/reactivity | Smoking index >0 | Yes vs no | Both cohorts; derived | Core; extended | Included for airway-injury relevance; derived from smoking index |
| Smoking index | Airway injury/reactivity | Pack-years/index | Continuous | Both cohorts; incomplete in derivation | Excluded | High derivation missingness and overlap with smoking history |
| Asthma | Airway injury/reactivity | Documented asthma | Yes vs no | Both cohorts | Core; extended | Clinically relevant airway-reactivity marker |
| Chronic bronchitis | Airway injury/reactivity | Documented chronic bronchitis | Yes vs no | Both cohorts | Extended only | Diagnosis-label variable assessed for incremental value |
| Albumin | Hematologic/nutritional status | Serum albumin, g/L | Per 5 g/L | Both cohorts | Core; extended | Objective laboratory marker available across centers |
| Hemoglobin | Hematologic | Hemoglobin, g/L | Per 10 g/L | Both cohorts | Core; extended | Objective hematologic marker available across centers |
| log10(BNP/NT-proBNP+1) | Cardiopulmonary burden | BNP or NT-proBNP | Log10 transformed | Both cohorts | Core; extended | Cardiopulmonary marker; transformation addressed skewness |
| CRP | Inflammatory | C-reactive protein | Per 10 units | Derivation only | Excluded | Not collected consistently in validation cohort |
| WBC | Inflammatory | White blood cell count | Continuous | Derivation only | Excluded | Not collected consistently in validation cohort |
| NLR | Inflammatory | Neutrophil-to-lymphocyte ratio | Continuous | Derivation only | Excluded | Not collected consistently in validation cohort |
| Pseudomonas aeruginosa | Microbiological | Documented isolation | Yes vs no | Derivation only | Excluded | Unavailable in validation cohort |
| Multilobar involvement | Structural/vascular burden | Multiple lobes involved | Yes vs no | Derivation only | Excluded | Unavailable in validation cohort |
| Bronchiectasis extent | Structural/vascular burden | Bronchiectatic bronchi count | Continuous | Derivation only; sparse | Excluded | High missingness and unavailable in validation cohort |
| PA pressure | Cardiopulmonary burden | Pulmonary artery pressure | Continuous | Derivation only; sparse | Excluded | High missingness and unavailable in validation cohort |
| Prior TB/TB sequelae | Etiologic/structural context | Incompletely ascertained in derivation cohort; unavailable in validation cohort | Yes vs no | Derivation only | Excluded | Absence of documentation could not be distinguished from true absence |
| Detailed morphology/vascular imaging | Structural/vascular burden | Cystic morphology, CTA/arterial features | Not standardized | Not consistently available | Excluded | Insufficient inter-center availability |
| BSI/FACED components | Severity | FEV1%, mMRC, exacerbation history, etc. | Score-specific | Incomplete | Excluded | Complete scores could not be calculated |

Clinical domains were prespecified solely to organize and interpret routinely collected variables; they do not represent data-derived phenotypes. No clustering, latent-class analysis, molecular profiling, or formal phenotyping was performed. BE, bronchiectasis; BNP, B-type natriuretic peptide; NT-proBNP, N-terminal pro-B-type natriuretic peptide; CRP, C-reactive protein; WBC, white blood cell count; NLR, neutrophil-to-lymphocyte ratio; PA, pulmonary artery; TB, tuberculosis; CTA, computed tomography angiography; BSI, Bronchiectasis Severity Index; FACED, FEV1, age, chronic colonization, extension, and dyspnea.

**Supplementary Table S2. Availability, missingness, and handling of candidate predictors.**

| **Variable** | **Derivation availability/**  **missingness** | **Validation availability/**  **missingness** | **Missing-data handling** | **Core model** | **External validation** |
| --- | --- | --- | --- | --- | --- |
| Age | 0 (0.0%) | 0 (0.0%) | No imputation required | Yes | Yes |
| Male sex | 0 (0.0%) | 0 (0.0%) | No imputation required in either cohort | Yes | Yes |
| Smoking history | Source smoking-index documentation unavailable for 114 patients (53.0%); the exported binary field contained no analytical missing values | 0 (0.0%) | Defined as smoking index >0. In the exported derivation dataset, unavailable source smoking-index values had been coded as 0; therefore, undocumented smoking status could not be distinguished from no recorded smoking history. No additional imputation was performed. | Yes | Yes |
| Smoking index | 114 (53.0%) | 0 (0.0%) | Not entered as a continuous predictor in the primary logistic models. Not entered into the primary logistic regression or exploratory machine-learning models. | No | No |
| Asthma | 0 (0.0%) after diagnosis coding | 0 (0.0%) | Absence of a documented diagnosis coded as 0 | Yes | Yes |
| Chronic bronchitis | 0 (0.0%) after diagnosis coding | 0 (0.0%) | Absence of a documented diagnosis coded as 0 | No | Extended only |
| Albumin | 5 (2.3%) | 0 (0.0%) | Median imputation in derivation; observed values in validation | Yes | Yes |
| Hemoglobin | 0 (0.0%) | 0 (0.0%) | No imputation required | Yes | Yes |
| BNP/NT-proBNP | 55 (25.6%) | 0 (0.0%) | Median imputation in derivation; log10(value + 1) transformation; observed values in validation | Yes | Yes |
| CRP | 22 (10.2%) | Not collected | Median imputation for derivation exploratory analysis | No | No |
| WBC | 1 (0.5%) | Not collected | Median imputation for derivation exploratory analysis | No | No |
| NLR | 0 (0.0%) | Not collected | Median if missing; none required | No | No |
| Pseudomonas aeruginosa | 0 (0.0%) | Not collected | Mode if missing; none required | No | No |
| Multilobar involvement | 0 (0.0%) after imaging coding | Not collected | Binary imaging abstraction | No | No |
| Bronchiectasis extent | 142 (66.0%) | Not collected | Excluded because of high missingness | No | No |
| PA pressure | 167 (77.7%) | Not collected | Excluded because of high missingness | No | No |
| Prior TB/TB sequelae | Not reliably ascertainable | Not collected | Excluded from formal analysis; no imputation performed | No | No |

Missingness was assessed before imputation. Derivation-cohort primary analyses used the exported single-imputed analytical dataset, whereas the percentages in this table describe the pre-imputation source data. Albumin and BNP/NT-proBNP were median-imputed; male sex required no imputation. Smoking history and smoking index are reported separately because the analytical smoking-history field contained no missing values after unavailable source smoking-index records had previously been coded as 0. Consequently, undocumented smoking status could not be distinguished from an explicitly recorded absence of smoking history. No final-model predictor required imputation in the external validation cohort. Exploratory machine-learning analyses used fold-specific imputation within the derivation cohort to prevent information leakage.

**Supplementary Table S3. Availability of components required for BSI and FACED calculation.**

| **Component** | **Required by** | **Derivation cohort** | **Validation cohort** | **Implication** |
| --- | --- | --- | --- | --- |
| Age | BSI; FACED | Available | Available | Usable |
| FEV1% predicted | BSI; FACED | Not systematically available | Not collected | Precluded both scores |
| mMRC dyspnea | BSI; FACED | Not systematically available | Not collected | Precluded both scores |
| Prior exacerbations/hospitalizations | BSI | Not systematically available | Not collected | Precluded BSI |
| Body mass index | BSI | Available with 12/215 missing | Not collected | Precluded external BSI |
| Pseudomonas colonization | BSI; FACED | Admission microbiology available; chronic colonization not reliably ascertainable | Not collected | Precluded external scores |
| Other chronic colonization | BSI | Incomplete | Not collected | Precluded BSI |
| Radiological extent | BSI; FACED | Partially available | Not collected | Precluded external scores |
| Overall score | BSI; FACED | Not fully evaluable | Not evaluable | AUC/DeLong comparison not performed |

A head-to-head AUC comparison with BSI and FACED was not performed because the components required to calculate complete scores were unavailable for a sufficient number of patients, particularly in the external validation cohort. No score component was imputed solely to construct BSI or FACED.

**Supplementary Table S4. Serum albumin distribution and prevalence of hypoalbuminemia according to hemoptysis status.**

| **Albumin measure** | **Hemoptysis** | **No hemoptysis** | **Overall** | **P value** |
| --- | --- | --- | --- | --- |
| Available albumin values, n | 53 | 157 | 210 | NA |
| Mean +/- SD, g/L | 38.9 +/- 5.5 | 35.7 +/- 5.3 | 36.5 +/- 5.5 | <0.001 |
| Median (IQR), g/L | 39.7 (36.9-41.1) | 36.3 (32.0-40.1) | 37.3 (32.5-40.3) | <0.001 |
| Albumin >=35 g/L, n (%) | 43 (81.1%) | 91 (58.0%) | 134 (63.8%) | 0.003 |
| Hypoalbuminemia (<35 g/L), n (%) | 10 (18.9%) | 66 (42.0%) | 76 (36.2%) | 0.003 |

Albumin values are based on observed derivation-cohort measurements before imputation. Hypoalbuminemia was operationally defined as serum albumin <35 g/L because laboratory reference intervals were not uniformly retained in the retrospective dataset. Continuous distributions were compared using the Mann-Whitney U test; categorical proportions were compared using Fisher's exact test. The association should not be interpreted as causal.

**Supplementary Table S5. Multicollinearity diagnostics for predictors in the final models.**

| **Model** | **Predictor** | **VIF** | **Tolerance** | **Maximum VIF** |
| --- | --- | --- | --- | --- |
| Core model | Age | 1.15 | 0.866 | 1.71 |
| Core model | Male sex | 1.71 | 0.586 | 1.71 |
| Core model | Smoking history | 1.46 | 0.683 | 1.71 |
| Core model | Asthma | 1.03 | 0.969 | 1.71 |
| Core model | Albumin | 1.47 | 0.679 | 1.71 |
| Core model | Hemoglobin | 1.63 | 0.612 | 1.71 |
| Core model | log10(BNP/NT-proBNP+1) | 1.21 | 0.826 | 1.71 |
| Bronchitis-extended model | Age | 1.21 | 0.824 | 1.72 |
| Bronchitis-extended model | Male sex | 1.72 | 0.580 | 1.72 |
| Bronchitis-extended model | Smoking history | 1.47 | 0.679 | 1.72 |
| Bronchitis-extended model | Asthma | 1.04 | 0.963 | 1.72 |
| Bronchitis-extended model | Chronic bronchitis | 1.09 | 0.918 | 1.72 |
| Bronchitis-extended model | Albumin | 1.48 | 0.678 | 1.72 |
| Bronchitis-extended model | Hemoglobin | 1.66 | 0.603 | 1.72 |
| Bronchitis-extended model | log10(BNP/NT-proBNP+1) | 1.23 | 0.816 | 1.72 |

VIF values were calculated from the derivation-cohort design matrix using the same coding and transformations as the logistic regression models. VIF, variance inflation factor; tolerance, 1/VIF.

**Supplementary Table S6. Complete logistic regression coefficients and model formulas.**

| **Model** | **Variable** | **Coding/scaling** | **beta** | **SE** | **OR** | **95% CI** | **P value** |
| --- | --- | --- | --- | --- | --- | --- | --- |
| Core model | Intercept | 1 | -4.077 | 2.141 | 0.02 | 0.00-1.13 | 0.057 |
| Core model | Age | Age/10 | -0.207 | 0.126 | 0.81 | 0.64-1.04 | 0.100 |
| Core model | Male sex | Male | 0.656 | 0.445 | 1.93 | 0.80-4.61 | 0.141 |
| Core model | Smoking history | SmokingHistory | 0.098 | 0.496 | 1.10 | 0.42-2.92 | 0.844 |
| Core model | Asthma | Asthma | -1.777 | 0.565 | 0.17 | 0.06-0.51 | 0.002 |
| Core model | Albumin | Albumin/5 | 0.666 | 0.211 | 1.95 | 1.29-2.94 | 0.002 |
| Core model | Hemoglobin | Hemoglobin/10 | -0.081 | 0.111 | 0.92 | 0.74-1.15 | 0.463 |
| Core model | log10(BNP/NT-proBNP+1) | log10(BNP/NT-proBNP+1) | 0.132 | 0.287 | 1.14 | 0.65-2.00 | 0.645 |
| Bronchitis-extended model | Intercept | 1 | -4.635 | 2.200 | 0.01 | 0.00-0.72 | 0.035 |
| Bronchitis-extended model | Age | Age/10 | -0.142 | 0.131 | 0.87 | 0.67-1.12 | 0.280 |
| Bronchitis-extended model | Male sex | Male | 0.530 | 0.455 | 1.70 | 0.70-4.14 | 0.244 |
| Bronchitis-extended model | Smoking history | SmokingHistory | 0.127 | 0.506 | 1.13 | 0.42-3.06 | 0.802 |
| Bronchitis-extended model | Asthma | Asthma | -1.852 | 0.569 | 0.16 | 0.05-0.48 | 0.001 |
| Bronchitis-extended model | Chronic bronchitis | ChronicBronchitis | -0.885 | 0.433 | 0.41 | 0.18-0.96 | 0.041 |
| Bronchitis-extended model | Albumin | Albumin/5 | 0.690 | 0.217 | 1.99 | 1.30-3.05 | 0.002 |
| Bronchitis-extended model | Hemoglobin | Hemoglobin/10 | -0.046 | 0.114 | 0.96 | 0.76-1.19 | 0.685 |
| Bronchitis-extended model | log10(BNP/NT-proBNP+1) | log10(BNP/NT-proBNP+1) | 0.056 | 0.292 | 1.06 | 0.60-1.87 | 0.848 |

Logistic regression models were fitted in the derivation cohort by maximum likelihood estimation using an iteratively reweighted least-squares algorithm. OR, odds ratio; CI, confidence interval; SE, standard error. The intercept is included to permit independent calculation of predicted probabilities.

Core model: logit(P) = -4.0772 - 0.2070 × Age/10 + 0.6560 × Male + 0.0976 × SmokingHistory - 1.7768 × Asthma + 0.6655 × Albumin/5 - 0.0814 × Hemoglobin/10 + 0.1321 × log10(BNP/NT-proBNP+1)

Bronchitis-extended model: logit(P) = -4.6347 - 0.1416 × Age/10 + 0.5302 × Male + 0.1266 × SmokingHistory - 1.8518 × Asthma - 0.8851 × ChronicBronchitis + 0.6900 × Albumin/5 - 0.0460 × Hemoglobin/10 + 0.0559 × log10(BNP/NT-proBNP+1)

For either model, predicted probability P = exp(logit(P)) / [1 + exp(logit(P))]. Binary predictors are coded as 1 when present and 0 when absent.

**Supplementary Table S7. Bootstrap internal validation of the core model.**

| **Model** | **Events** | **Predictor parameters** | **EPV** | **Apparent AUC** | **Bootstrap optimism** | **Optimism-corrected AUC** | **Corrected calibration slope** |
| --- | --- | --- | --- | --- | --- | --- | --- |
| Core model | 55 | 7 | 7.9 | 0.747 | 0.040 | 0.707 | 0.786 |

Internal validation used 1,000 successful bootstrap resamples (seed 20260801). EPV was calculated as the number of hemoptysis events divided by the number of predictor parameters, excluding the intercept. The analysis used the same single-imputed derivation dataset as model development. With 55 events and seven predictor parameters (EPV 7.9), residual overfitting and coefficient instability remain possible despite bootstrap correction; these estimates should therefore be considered preliminary.

**Supplementary Table S8. Detailed external risk stratification based on model-predicted tertiles.**

| **Model** | **Risk stratum** | **n** | **Events** | **Observed hemoptysis** | **Mean predicted risk** |
| --- | --- | --- | --- | --- | --- |
| Core model | Low | 35 | 1 | 2.9% | 21.4% |
| Core model | Intermediate | 35 | 12 | 34.3% | 35.7% |
| Core model | High | 35 | 23 | 65.7% | 51.0% |
| Bronchitis-extended model | Low | 35 | 5 | 14.3% | 20.7% |
| Bronchitis-extended model | Intermediate | 35 | 13 | 37.1% | 37.6% |
| Bronchitis-extended model | High | 35 | 18 | 51.4% | 53.6% |

Risk strata were defined separately for each model using tertiles of predicted risk in the Shijiazhuang People's Hospital external validation cohort.

**Supplementary Table S9. External calibration metrics and recalibration analyses.**

| **Model** | **Observed event rate** | **Mean predicted** | **Calibration intercept (95% CI)** | **Calibration slope (95% CI)** | **Brier score** | **Intercept update** | **Updated Brier** | **Recalibrated Brier** |
| --- | --- | --- | --- | --- | --- | --- | --- | --- |
| Core model | 34.3% | 36.0% | 0.725 (0.012–1.438) | 2.773 (1.513–4.033) | 0.177 | -0.084 | 0.178 | 0.163 |
| Bronchitis-extended model | 34.3% | 37.3% | 0.134 (−0.430–0.698) | 1.688 (0.787–2.589) | 0.187 | -0.146 | 0.186 | 0.183 |

Calibration intercepts and slopes were estimated by logistic recalibration of the external outcome on the logit of the original predicted probability and are reported with 95% confidence intervals. Ideal values are 0 for the calibration intercept and 1 for the calibration slope. Intercept-only updating re-estimated the intercept with the original linear predictor entered as an offset. The fully recalibrated Brier score was obtained after re-estimating both the intercept and slope in the validation cohort and should be regarded as descriptive rather than as an independent validation estimate.

**Supplementary Table S10. Hyperparameter tuning and internal validation of the exploratory machine-learning models.**

| **Item** | **Random forest** | **Gradient boosting** |
| --- | --- | --- |
| Analysis role | Exploratory secondary analysis | Exploratory secondary analysis |
| Analysis cohort | Derivation cohort only | Derivation cohort only |
| Sample size/events | 215/55 | 215/55 |
| Input predictors | 15 predictors listed in Supplementary Methods | Same 15 predictors |
| Missing-data handling | Training-fold median/mode imputation | Training-fold median/mode imputation |
| Transformation | log10(BNP/NT-proBNP + 1); no standardization | Same |
| Class imbalance | Inverse-frequency class weights | Inverse-frequency observation weights |
| Tuning method | Grid search | Grid search |
| Outer validation | Stratified 5-fold CV x 5 repeats | Stratified 5-fold CV x 5 repeats |
| Inner tuning | Stratified 4-fold CV | Stratified 4-fold CV |
| Optimization metric | ROC AUC | ROC AUC |
| Search space | ntree: 500; mtry: 2, 4, 6; nodesize: 3, 8 | n.trees: 150, 300, 600; depth: 1, 2; shrinkage: 0.01, 0.05; minimum node size: 5, 10; bag fraction: 0.8 |
| Selected hyperparameters | ntree=500; mtry=2; nodesize=3 | n.trees=150; interaction.depth=2; shrinkage=0.01; n.minobsinnode=10; bag.fraction=0.8 |
| Internally validated AUC | 0.731 (SD 0.053) | 0.671 (SD 0.081) |
| External validation | Not performed | Not performed |

AUC values are the mean (SD) across 25 outer validation folds. Hyperparameters were optimized independently within each outer training set; the selected hyperparameters shown are those obtained from the final four-fold tuning run on the full derivation cohort for reproducible exploratory refitting. CV, cross-validation; AUC, area under the receiver operating characteristic curve.

**Supplementary Table S11. Permutation feature-importance values from the exploratory machine-learning models.**

| **Predictor** | **Random forest mean (SD)** | **RF rank** | **Gradient boosting mean (SD)** | **GB rank** |
| --- | --- | --- | --- | --- |
| Asthma | 0.0726 (0.0198) | 1 | 0.0657 (0.0205) | 1 |
| Albumin | 0.0427 (0.0281) | 2 | 0.0594 (0.0653) | 2 |
| White blood cell count | 0.0183 (0.0196) | 3 | 0.0142 (0.0249) | 3 |
| Chronic bronchitis | 0.0174 (0.0127) | 4 | 0.0054 (0.0091) | 5 |
| C-reactive protein | 0.0138 (0.0192) | 5 | 0.0083 (0.0161) | 4 |
| Pulmonary artery pressure | 0.0086 (0.0145) | 6 | 0.0018 (0.0100) | 6 |
| Smoking history | 0.0067 (0.0087) | 7 | -0.0000 (0.0000) | 9 |
| COPD | 0.0054 (0.0105) | 9 | 0.0000 (0.0039) | 8 |
| Age | 0.0059 (0.0197) | 8 | -0.0012 (0.0189) | 10 |
| Multilobar involvement | 0.0046 (0.0132) | 10 | -0.0016 (0.0041) | 11 |
| Male sex | 0 (0.0%) | 12 | No imputation required in either cohort | 7 |
| log10(BNP/NT-proBNP + 1) | 0.0033 (0.0180) | 11 | -0.0080 (0.0216) | 14 |
| Hemoglobin | -0.0000 (0.0147) | 13 | -0.0071 (0.0121) | 13 |
| Bronchiectasis extent | -0.0053 (0.0130) | 15 | -0.0028 (0.0050) | 12 |
| Neutrophil-to-lymphocyte ratio | -0.0045 (0.0207) | 14 | -0.0085 (0.0209) | 15 |

Importance was defined as the decrease in held-out validation AUC after 20 permutations of each predictor within each outer fold and was summarized across 25 outer folds. SD denotes the between-fold SD of fold-level mean importance. Negative values indicate no reproducible improvement in discrimination under permutation. Ranks are based on mean importance. RF, random forest; GB, gradient boosting.

**Supplementary Figure S1. Pearson correlation heatmap of the final-model predictors.**

The heatmap shows pairwise Pearson correlations among the predictors assessed in the derivation cohort. This analysis was used as an auxiliary assessment of collinearity and should be interpreted together with the VIF results in Supplementary Table S5.

**Supplementary Figure S2. Spearman correlation heatmap of the final-model predictors.**

The heatmap shows pairwise Spearman rank correlations among the predictors assessed in the derivation cohort as a non-parametric sensitivity analysis. The results should be interpreted together with the VIF analysis in Supplementary Table S5.

**Supplementary Figure S3. Calibration of the bronchitis-extended model in the external validation cohort.**

Calibration was evaluated by comparing observed hemoptysis rates with mean predicted probabilities across quintiles of predicted risk. The diagonal line represents ideal agreement. The bronchitis-extended model showed imperfect calibration and should be interpreted together with its discrimination and risk-stratification results.

**Supplementary Figure S4. Risk stratification by the bronchitis-extended model in the external validation cohort.**

Patients were divided into low-, intermediate-, and high-risk groups according to tertiles of model-predicted risk. Bars show observed hemoptysis rates and mean predicted probabilities within each stratum.

**Supplementary Figure S5. Feature importance in exploratory random forest and gradient boosting models.**

Panel A shows the random forest model and panel B the gradient boosting model. Both models were developed and evaluated exclusively in the derivation cohort (n = 215; 55 hemoptysis events) using nested stratified five-fold cross-validation repeated five times. AUC values are the mean (SD) across 25 held-out outer validation folds. Bars show cross-validated permutation importance, defined as the mean decrease in validation AUC after 20 permutations per predictor within each outer fold. All 15 input variables, including smoking history, are displayed. Negative values indicate no reproducible improvement in discrimination under permutation. Feature importance reflects exploratory predictive associations and should not be interpreted as regression coefficients, causal effects, or externally validated evidence.

**Supplementary Data, Code, and Reporting Checklist**

The final de-identified analytical datasets used for model development and external validation are provided as Supplementary Data S1 and Supplementary Data S2, respectively. Complete R code is provided as Supplementary Code S1. The code reproduces data preprocessing, missing-data handling, descriptive analyses, logistic-regression model development, bootstrap internal validation, external validation, calibration assessment, recalibration, decision-curve analysis, risk stratification, correlation analyses, variance-inflation diagnostics, nomogram construction, and exploratory machine-learning analyses. Package versions, random seeds, file definitions, and execution instructions are documented in the accompanying README file. The completed TRIPOD+AI checklist is provided as Supplementary Checklist S1.

**Supplementary Data S1.** Final de-identified derivation analytical dataset (n = 215).

**Supplementary Data S2.** Final de-identified external-validation analytical dataset (n = 105).

**Supplementary Code S1.** Complete R code for all reported statistical analyses.

**Supplementary Checklist S1.** Completed TRIPOD+AI reporting checklist.
